# Epidemiology, clinical characteristics, and risk factors of SARS-CoV-2 in Côte d'Ivoire: a cross-sectional study

**DOI:** 10.11604/pamj.2026.53.26.47947

**Published:** 2026-01-19

**Authors:** Jacques Danho Monney, Herve Alberic Kadjo, Yakoura Karidja Ouattara, Valery Edgard Adjogoua, Daouda Coulibaly, Mireille Dosso, Meité Syndou, Karamoko Yahaya

**Affiliations:** 1 Biological Sciences Training and Research Unit, Animal Biology Department, Peleforo Gon Coulibaly University, Korhogo, Côte d’Ivoire,; 2 Pasteur Institute, Abidjan, Côte d'Ivoire,; 3 National Institute of Public Hygiene, Abidjan, Côte d'Ivoire,; 4 Nature Sciences Training and Research Unit, Animal Biology and Cytology Laboratory, Nangui Abrogoua University, Abidjan, Côte d'Ivoire

**Keywords:** COVID-19, epidemiology, risk factors, clinical characteristics, SARS-CoV-2, Côte d’Ivoire

## Abstract

**Introduction:**

the COVID-19 pandemic has posed significant epidemiological challenges globally, with marked regional variations in transmission, severity, and outcomes. In Côte d'Ivoire, disparities in healthcare infrastructure, testing capacity, and demographics have influenced SARS-CoV-2 infection dynamics. This study aims to describe the epidemiological and clinical characteristics of SARS-CoV-2 infection, as well as to identify the risk factors associated with the severity of the infection.

**Methods:**

a study was conducted using nasopharyngeal samples collected between January and December 2020, as part of Côte d'Ivoire's national COVID-19 response. The study included individuals from all socio-professional groups across urban and rural areas. Sampling was performed in various settings, including hospitals, testing centres, and community-based screenings. The testing was voluntary, systematic, and based on several factors, including both symptomatic and asymptomatic individuals, contacts of confirmed cases, suspected individuals, hospitalised patients, and participation in public health campaigns. All individuals who underwent testing were included without any specific selection process. The study population was generally representative of the national population, although a higher proportion of urban individuals were included due to limited testing in rural areas. SARS-CoV-2 was detected by real-time RT-PCR. Statistical analyses included chi-squared tests, logistic regression, and Multiple Correspondence Analysis (MCA) to identify factors associated with positivity, hospitalisation, and mortality.

**Results:**

the study included 240,599 individuals, with a mean age of 38 years (SD = 13.97). Of the total, 93,391 (39%) were women and 147,208 (61%) were men. The SARS-CoV-2 prevalence was 9.31% (95% CI: [0.89 - 0.97]), with higher prevalence in women (10.07%) compared to men (8.83%) (X^2^ = 102.47, df = 1, P < .001). Positivity rate varied by age, ranging from 5.76% in children (0-5 years) to 11.13% in the elderly (60+ years) (X^2^ = 233.41, df = 5, P < .001). In multivariable analysis of hospitalisation, each additional year of age increased the probability of hospitalisation by 2.3% (aOR = 1.023, 95% CI: [1.014 - 1.032], P < .001). Patients with cough were 4.15 times more likely to be hospitalised (aOR = 4.15, 95% CI: [3.18 - 5.39], P < .001). Gastrointestinal symptoms (diarrhoea or vomiting) increased the likelihood of hospitalisation by 367 times (aOR = 367, 95% CI: [68.01 - 1686.00], P < .001). For mortality, each additional year of age increased mortality risk by 16% (aOR = 1.16, 95% CI: [1.15 - 1.16], P < .001). Patients with cough had 64.67 times higher risk of death (aOR = 64.67, 95% CI: [62.92 - 66.47], P < .001).

**Conclusion:**

this study identifies key factors influencing hospitalisation and mortality in SARS-CoV-2-positive patients. Age, cough, and gastrointestinal symptoms (diarrhoea or vomiting) were strongly associated with an increased likelihood of hospitalisation, while age and cough were significant predictors of mortality. These findings highlight the importance of early identification and monitoring of patients with these characteristics, particularly in settings with limited healthcare infrastructure.

## Introduction

The COVID-19 pandemic, caused by the SARS-CoV-2 virus, was first identified in December 2019 in Wuhan, China, and rapidly spread worldwide, severely impacting healthcare systems, economies, and societies [1]. COVID-19, which causes respiratory infections ranging from mild symptoms to severe forms, including pneumonia and acute respiratory distress syndrome, represents a major public health threat [2]. Globally, the pandemic has affected over 216 countries, leading to millions of confirmed cases and hundreds of thousands of deaths [3]. The World Health Organization (WHO) declared COVID-19 a global pandemic in March 2020, emphasising the urgent need to curb its spread [4].

In Africa, although the number of reported COVID-19 cases has been lower compared to other regions, the pandemic has exposed significant vulnerabilities within healthcare systems. These include inadequate infrastructure and limited access to medical services, which may have affected the reporting and management of cases [5]. Epidemiological studies have shown that some African countries, particularly in West Africa, have experienced lower mortality rates, potentially due to factors such as a younger population and early containment measures [6]. However, there are considerable disparities in pandemic management across West Africa. Countries such as Nigeria have reported higher prevalence and mortality rates, whereas others have managed to control transmission more effectively. These differences underscore the need for a comprehensive analysis of the epidemiological and clinical factors specific to this region to improve screening, prevention, and case management. While common risk factors for SARS-CoV-2 have been identified globally, including age, comorbidities, and certain symptoms [7]; transmission is particularly complex in West Africa, where testing and contact tracing mechanisms are limited.

In Côte d´Ivoire, the situation has been challenged by the lack of healthcare infrastructure in certain rural areas and limited testing capacities. This has led to underreporting of cases, making it difficult to accurately assess the true scale of the pandemic. In such a context, many asymptomatic infections went unnoticed. The absence of comprehensive data on the epidemiological and clinical characteristics of SARS-CoV-2 has left many questions unanswered, particularly regarding the prevalence of asymptomatic cases and specific risk factors. This study aims to describe the epidemiological and clinical characteristics of SARS-CoV-2 infection in this setting, as well as to identify the key risk factors associated with severe outcomes. These findings could strengthen epidemiological surveillance and improve screening strategies, healthcare responses, and management of future epidemics, particularly in rural areas.

## Methods

**Study design and setting:** this study is a cross-sectional study conducted as part of the national response to the COVID-19 pandemic in Côte d'Ivoire, from January to December 2020. Samples and data were collected from various healthcare facilities, including hospitals, testing centres, community-based screenings, health districts and homes across urban and rural areas. The samples were processed at the Department of Epidemic Viruses (DVE) at the Pasteur Institute of Abidjan, where the molecular diagnosis of SARS-CoV-2 was carried out.

**Study population:** this study included individuals from all socio-professional groups, with no specific inclusion or exclusion criteria based on clinical status, comorbidities, or epidemiological factors. The study population comprised hospitalised patients, confirmed COVID-19 cases, contacts of confirmed cases, and suspected cases, regardless of whether they were symptomatic or asymptomatic. The sample size was determined by the large-scale data collection undertaken as part of the national response to the COVID-19 pandemic. The sampling process aimed to be representative of the national population, including both urban and rural areas. Nasopharyngeal swabs were performed by agents of the National Institute of Public Hygiene (INHP), who were mandated by health authorities to collect and transport the samples to the DVE.

**Data collection:** a structured questionnaire was used to collect information on symptoms, clinical data, demographic information, and potential risk factors. This questionnaire was administered by agents from the INHP using electronic tablets to record responses before the biological sample collection. The data was recorded electronically in real-time through the ODK Collect (v2020.3.1) application. Nasopharyngeal swabs were collected, stored at +4°C before being transported to the DVE. Viral RNA extraction was performed manually using the QIAamp® Viral RNA Mini Kit (QIAGEN, Cat. No. 52904, Germany), and SARS-CoV-2 was detected using a multiplex RT-qPCR assay with the STANDARD M nCoV Real-Time Detection Kit (SD BIOSENSOR, Ref. M-NCOV-01, Korea). Amplifications were conducted on a QuantStudio 5 thermocycler (Thermo Fisher Scientific, USA), and the results were analysed using the Design and Analysis Software 2.6.0 (Thermo Fisher Scientific, USA) for PCR data interpretation. The data collected through the survey, along with the PCR results, were entered into an Excel database, ensuring systematic and accurate data collection and management. All information was securely stored with restricted access to ensure confidentiality and data integrity.

### Definitions

**Symptomatic:** a participant is considered symptomatic if, at the time of sample collection, they present one or more clinical symptoms commonly associated with COVID-19, such as cough, fever, shortness of breath, fatigue, etc. Asymptomatic**:** a participant is considered asymptomatic if they test positive for SARS-CoV-2, but do not show any clinical symptoms at the time of sample collection. Confirmed COVID-19 cases**:** a confirmed case is a participant whose SARS-CoV-2 infection has been confirmed by a positive PCR test. Contacts of confirmed cases: a contact of a confirmed case is an individual who has had close contact with a person confirmed to be positive for COVID-19, particularly within the 14 days before sample collection. Suspected cases: a suspected case is a participant showing clinical symptoms compatible with COVID-19 (such as cough, fever, shortness of breath) or having had recent contact with a confirmed COVID-19 positive case, but without confirmation by PCR testing. SARS-CoV-2 positive: a participant is considered SARS-CoV-2 positive if their PCR test for SARS-CoV-2 has confirmed the presence of the virus RNA in a nasopharyngeal sample. Risk factors: conditions or characteristics associated with an increased risk of severe COVID-19 infection. This includes variables such as age, gender, comorbidities (e.g., hypertension, diabetes), and other symptoms such as cough or diarrhoea.

**Statistical analysis:** the database was analysed using R-4.4.2 (R Studio Version 2024.12.0+467) with both descriptive and inferential statistical methods. Differences between groups were assessed using the Chi-square test (X^2^), with p-values < 0.05 indicating statistical significance. Logistic regression analyses were performed, starting with univariable regressions to examine each variable independently, followed by multivariable regressions to adjust for confounding factors and obtain adjusted odds ratios (aOR) with 95% confidence intervals (CI). Collinear variables were combined into composite variables to preserve model integrity and avoid missing values (NA). The final multivariable model included all significant variables from the univariable analyses (p < 0.05), adjusting for factors such as age and symptoms (e.g., cough, diarrhoea). These tests were performed with a significance level set at p < 0.05, and results were interpreted considering adjusted odds ratios and confidence intervals to assess the strength and precision of associations. Multiple Correspondence Analysis (MCA) was used for multivariate stratification of SARS-CoV-2 positive and negative cases, exploring the relationship between symptoms, positivity, and risk factors. Missing data were addressed by imputation, replacing missing values with the mean of the respective variables to minimise bias.

**Ethical considerations:** this study was conducted using data collected through the national COVID-19 surveillance response system in Côte d'Ivoire, under the authority of the Ministry of Health and Public Hygiene. The analysis of these data was authorised by the ministry and did not require specific approval from the national ethics committee due to the public health emergency context. All procedures adhered to the ethical principles outlined in the Declaration of Helsinki and complied with the World Health Organization Regional Office for Africa (WHO-AFRO) protocol for the surveillance of influenza and other respiratory viruses.

## Results

**General characteristics of the study population:** the study included a total of 240,599 individuals, with a mean age of 38 years (SD = 13.97). Of these, 93,391 (39%) were women and 147,208 (61%) were men. The majority of the population, 86.38%, were asymptomatic, while 13.62% were symptomatic. The SARS-CoV-2 prevalence was 9.31% (95% CI: [0.89 - 0.97]), with higher prevalence in women (10.07%) compared to men (8.83%) (X^2^ = 102.47, df = 1, P <.001). Positivity rate varied by age, ranging from 5.76% in children (0-5 years) to 11.13% in the elderly (60+ years), with other age groups showing rates ranging from 7.44% to 9.3% (X^2^ = 233.41, df = 5, P <.001).

**Proportions of asymptomatic and symptomatic positive cases:** among the positive SARS-CoV-2 cases, 72.03% were asymptomatic, and 27.97% exhibited symptoms. This difference was statistically significant (X^2^ = 37.083, df = 1, P <.001).

**Factors associated with hospitalisation and mortality, univariate and multivariate logistic regression analysis:** the univariate logistic regression analysis showed that age, cough, diarrhoea, and vomiting were significantly associated with hospitalisation. Each additional year of age increased the probability of hospitalisation by 2.5% (OR = 1.025, (95% CI: [1.016 - 1.034]), P <.001), while patients with a cough were 4.30 times more likely to be hospitalised (OR = 4.30, (95% CI: [3.30 - 5.57]), P <.001). Diarrhoea and vomiting increased hospitalisation risk by 627 times (OR = 627.03, (95% CI: [128.01 - 2570.42]), P <.001). Factors like sex, sore throat, nasal discharge, shortness of breath, and respiratory difficulty were not significant (P > 0.05). In multivariable analysis, age, cough, and gastrointestinal symptoms (diarrhoea or vomiting) remained significant. Each year of age increased hospitalisation probability by 2.3% (aOR = 1.023, (95% CI: [1.014 - 1.032]), P <.001), and patients with cough were 4.15 times more likely to be hospitalised (aOR = 4.15, (95% CI: [3.18 - 5.39]), P <.001). Gastrointestinal symptoms increased hospitalisation likelihood by 367 times (aOR = 367, 95% CI: [68.01 - 1686.00]), P <.001). For mortality, univariate analysis found age (OR = 1.08, (95% CI: [1.08 - 1.09]), P <.001) and cough (OR = 12.65, (95% CI: [12.05 - 13.28]), P <.001) to be significant. Other factors showed no impact on mortality (P > 0.05). In multivariable analysis, each additional year of age increased mortality risk by 16% (aOR = 1.16, (95% CI: [1.15 - 1.16]), P <.001), and patients with cough had 64.67 times higher risk of death (aOR = 64.67, (95% CI: [62.92 - 66.47]), P <.001), as shown in Table 1.

**Table 1:** odds of factors associated with hospitalisation and mortality related to SARS-CoV-2 positivity

Factors	Factors-related hospitalisation			
	Unadjusted ORs (95% CI)	p-value	adjusted ORs (95% CI)	p-value
Age	1.025 (1.016 - 1.034)	<.001	1.023 (1.014 - 1.032)	<.001
Cough	4.30 (3.30 - 5.57)	<.001	4.15 (3.18 - 5.39)	<.001
Gastrointestinal (Diarrhoea or Vomiting)	627.03 (128.01 - 2570.42)	<.001	366.99 (68.01 - 1686.00)	<.001
	**factors-related mortality**			
Age	1.08 (1.08 - 1.09)	<.001	1.16 (1.15 - 1.16)	<.001
Cough	12.65 (12.05 - 13.28)	<.001	64.67 (62.92 - 66.47)	<.001

OR: odds ratio; aOR: adjusted odds ratio; CI: confidence interval; P < 0.05 considered statistically significant

**Figure 1:**
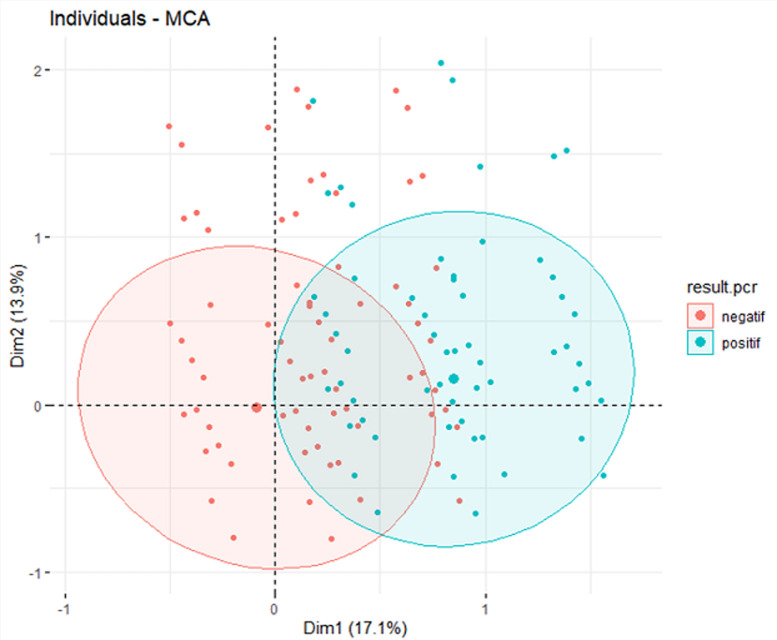
multiple correspondence analysis (MCA) plot of positive and negative SARS-CoV-2 cases

**Multivariate stratification of COVID-19 cases using multiple correspondence analysis (MCA):** MCA was used to explore associations between clinical and epidemiological variables and SARS-CoV-2 test outcomes. As shown in Figure 1, dimension 1, which accounted for 17.1% of the variance, effectively stratified positive and negative cases. Positive cases were predominantly on the right, associated with symptoms like cough, hospitalisation, contact with a confirmed case, and abnormal temperature. Negative cases clustered on the left, linked to the absence of symptoms and exposure. Dimension 2, explaining 13.9% of the variance, revealed greater clinical heterogeneity within the positive cases, showing a broader distribution (including asymptomatic carriers and severe cases). In contrast, negative cases had a more homogeneous clinical profile, reflected by tighter clustering along this axis.

## Discussion

The primary objective of this study was to identify the factors associated with hospitalisation and mortality in SARS-CoV-2-positive patients. The results highlighted three key determinants: age, respiratory symptoms (especially cough), and gastrointestinal symptoms (diarrhoea and vomiting), all of which were strongly associated with hospitalisation and mortality. In particular, age and cough were identified as significant predictors of mortality, while gastrointestinal symptoms were linked to hospitalisation risk. A notably high proportion of asymptomatic infections was also observed, raising concerns about the effectiveness of epidemiological surveillance and the control of community transmission.

Our findings confirm that age is a major risk factor, with each additional year increasing the probability of hospitalisation by 2.3% and the risk of mortality by 16%. This trend aligns with the findings of Riziki *et al*. [8], who reported in an African meta-analysis that older age multiplied the risk of death by 3.73. The similarity of results suggests that, regardless of geographic context, ageing remains a universal vulnerability factor in the face of COVID-19. This relationship could be explained by the progressive decline in immune response and the increased prevalence of chronic comorbidities (hypertension, diabetes, and cardiovascular diseases) among older individuals, which worsens the prognosis. In the Ivorian context, this observation is particularly concerning as the care of elderly patients remains limited, underscoring the need for targeted prevention strategies and enhanced clinical monitoring for this high-risk group. Cough, a hallmark symptom of respiratory infections, was also strongly associated with hospitalisation (aOR = 4.15) and mortality (aOR = 64.67). This relationship contrasts with the findings of Yupari-Azabache *et al*. [9], who identified productive cough as a protective factor (OR = 0.664). However, the divergence could be attributed to differences in epidemiological context and case definitions. In our study, cough was likely a clinical marker of severity, reflecting acute pulmonary inflammation requiring hospitalisation. This hypothesis is supported by Valladares-Garrido *et al*. [10], who showed that 84.72% of deceased patients had a cough at the time of admission, suggesting that cough is not a causal factor for mortality but rather an indicator of respiratory severity. Thus, in the context of our study, cough reflects a more severe clinical condition rather than an independent risk or protective factor.

Gastrointestinal symptoms (diarrhoea and vomiting) were also significantly associated with an increased risk of hospitalisation (aOR = 366.99), confirming their role as indicators of clinical severity. This finding echoes that of Khamis *et al*. [11], who reported a significant association between gastrointestinal symptoms and in-hospital mortality in COVID-19 patients in the Middle East. These manifestations may reflect a more extensive systemic viremia or direct involvement of the gastrointestinal system by SARS-CoV-2, a virus known to bind to ACE2 receptors expressed in the intestinal epithelium. These results highlight the need to integrate non-respiratory symptoms into clinical triage protocols, as they may precede or accompany severe forms of the disease. Another notable finding of this study was the high proportion of asymptomatic infections (72.03%), which aligns with the observations of Langat *et al*. [12] and Doumbia *et al*. [13], who reported that most SARS-CoV-2 infections in Africa were asymptomatic, particularly in community cohorts. Similarly, Ma *et al*. [14] estimated that 40.5% of all SARS-CoV-2 infections were asymptomatic, underscoring the significant role these silent carriers play in community transmission. In our context, this very high proportion may be linked to the relative youth of the Ivorian population and prior cross-immunity from exposure to other coronaviruses. These results emphasise the need to strengthen community-based screening, including for individuals without apparent symptoms, and to incorporate the surveillance of asymptomatic cases into control strategies to limit virus transmission.

These results have significant public health implications. Older patients, as well as those presenting persistent cough or gastrointestinal symptoms, should be prioritised for enhanced monitoring and early intervention, especially in healthcare settings. Early identification of these high-risk patients could allow for tailored management strategies, prevent severe complications, and reduce the burden on healthcare systems. Additionally, the high proportion of asymptomatic infections highlights the crucial role of silent carriers in community transmission. This emphasises the need to implement broader screening strategies, particularly for individuals without apparent symptoms. Furthermore, integrating the surveillance of asymptomatic cases into control strategies is crucial to limit virus transmission.

This study has several important strengths, including the use of a large and representative sample, allowing for a robust analysis of factors associated with hospitalisation and mortality. The statistical methods employed, such as multivariable logistic regression, enabled us to adjust for confounding effects and provide significant results. However, several limitations must be noted. The data were collected at a specific point in time during the pandemic, which may limit the generalisation of the results to other periods, especially with evolving virus variants. Additionally, while we adjusted for key confounders, other unmeasured variables, such as specific comorbidities, individual behaviours, or medical interventions, could have influenced the results. The analysis of mortality was marked by a significant imbalance between the number of deaths (very few) and non-deaths, which may have introduced bias into the logistic regression modelling.

## Conclusion

This study identifies key factors influencing hospitalisation and mortality in SARS-CoV-2-positive patients. Age, cough, and gastrointestinal symptoms (diarrhoea or vomiting) were strongly associated with an increased likelihood of hospitalisation, while age and cough were significant predictors of mortality. These findings highlight the importance of early identification and monitoring of patients with these characteristics to prevent severe outcomes. Furthermore, the high proportion of asymptomatic infections calls for enhanced surveillance and early detection strategies, especially in rural areas of Côte d'Ivoire. Establishing a robust system for epidemiological education and surveillance in both hospitals and communities will be crucial in controlling future outbreaks, limiting transmission, and ensuring a timely response in high-risk areas.

### What is known about this topic

- Age is a significant risk factor for COVID-19 severity;
- Respiratory symptoms like cough are commonly associated with severe outcomes;
- Asymptomatic infections contribute to virus transmission.

### What this study adds

- This study found that gastrointestinal symptoms strongly predict hospitalisation risk;
- A significant proportion of SARS-CoV-2 infections were asymptomatic (72.03%);
- Age and cough were identified as strong predictors of both hospitalisation and mortality.
